# Role of pharmacist in multidisciplinary pediatric intensive care rounds: a retrospective descriptive study

**DOI:** 10.1186/cc13193

**Published:** 2014-03-17

**Authors:** S Tripathi, K Graner, K Fryer, G Arteaga

**Affiliations:** 1 Mayo Clinic, Rochester, MN, USA

## Introduction

Multidisciplinary rounding practices in the ICU have now become the standard of care in most institutions. Few objective data are available, however, on the value each individual member of the team brings to the patient care. In our institution, pediatric pharmacists have been an integral part of the PICU rounds since 2002, although their role has evolved over the course of years. This study was undertaken with the primary aim of identifying the impact of pharmacist involvement in PICU rounds, with respect to changes in therapy.

## Methods

Since January 2003, pharmacists have recorded their clinical interventions and outcomes of those interventions in an institutionally developed Pharmaceutical Care database (P-Care). An intervention is defined as any recommendation the pharmacist makes to the patient care team regarding a change in the patient management or medication therapy. P-Care is designed to assist the pharmacist to optimize medication therapy, identify medication-related problems, decrease medication costs, improve pharmacist efficiency and document pharmacist workload. Data concerning pharmacists' interventions were extracted from the P-Care database in yearly increments via a reporting functionality that is available in the P-Care system. This study was exempted for review by the Mayo Clinic IRB.

## Results

From 1 January 2003 through 31 December 2012 pharmacists made 24,207 clinical interventions in the PICU and 19,252 of those interventions resulted in changes in medication therapy or therapy monitoring. Interventions that were accepted by the team involved 10,361 (53.8%) drug dosing regimen changes, 292 (1.5%) drug interactions or incompatibility, 969 (5%) drug monitoring suggestions, 1,665 (8.7%) drug routes/methods of administration, 3,895 (20.2%) drug selections, and 2,070 (10.8%) medication profile/order clarifications. As the pharmacist role on the PICU multidisciplinary rounds has evolved, the number of interventions has increased from 1,643 in 2003 to 3,799 in 2012. Of the 19,252 interventions that were implemented, 304 were deemed to be of potentially life-threatening consequences, 10,767 had a moderate impact, and 8,181 had minimal impact on patient outcome.

## Conclusion

To our knowledge this is the largest reported data on pharmacist's involvement in pediatric intensive care. This can serve as background knowledge on implementing measures on novel methodologies to integrate pharmacists in intensive care practice.

